# Increased beta-HFO phase-amplitude coupling in the subthalamic nucleus during movement in Parkinson's disease

**DOI:** 10.1016/j.ynirp.2026.100353

**Published:** 2026-05-13

**Authors:** András Puszta, Dénes Zádori, Péter Klivényi

**Affiliations:** a University of Szeged, Department of Neurology, Hungary; b Helgeland Hospital, Department of Neuropsychology, Norway; c University of Oslo, Department of Psychology, Norway; d HUN-REN-SZTE Neuroscience Research Group, Hungarian Research Network, University of Szeged, Hungary

**Keywords:** Parkinson's disease, Subthalamic nucleus, Local field potential, Phase-amplitude coupling, MDS-UPDRS

## Abstract

In Parkinson's disease (PD), increased amplitude of high-frequency oscillations (HFO) has been confirmed to be coupled with the beta oscillations phase, resulting in increased phase-amplitude coupling (PAC) in the subthalamic nucleus (STN). This pathological coupling correlates with the severity of motor symptoms and is known to be modulated by therapeutic interventions. For example, dopaminergic medications and deep brain stimulation (DBS) were previously shown to reduce PAC, which changes in magnitude between states of rest and movement. However, PAC alterations during kinetic and static movements in the presence and absence of medications remain to be determined. Furthermore, there is little evidence on the relationship between PAC and clinical symptoms of PD. In this study, we investigated these two issues. We analyzed a publicly available dataset, which contained STN local field potential registrations (n = 20) and concurrent limb electromyography during resting, static, and kinetic movements with and without levodopa intake. We calculated the PAC within the STN during different conditions, and the PAC between the limb tremor and the ipsilateral LFP oscillations. Beta-HFO STN PAC was increased during the medication off period, especially during kinetic movement and was highly correlated with bradykinesia and negatively correlated with tremor. We observed no interaction between tremor frequency oscillation phase on electromyography and beta or HFO oscillation amplitude within the STN in either of the conditions. These results point out that beta-HFO PAC provides complementary information to beta power for electrophysiological localization during DBS implantation and during adaptive DBS.

## 1 Introduction

Deep brain stimulation (DBS) has emerged as a powerful therapeutic tool to alleviate motor symptoms and improve quality of life of patients with Parkinson's disease (PD) (Benabid et al., 2009). This success has led to extensive research on the electrophysiological processes in the subthalamic nucleus (STN), which us a key basal ganglia structure implicated in the pathophysiology of PD (Hamani et al., 2004). Early investigations focused on local field potential (LFP) power within the STN and revealed higher beta (13–30 Hz) and gamma (30–100 Hz) oscillations in patients with PD than in healthy controls (Jenkinson et al., 2013; Kuhn et al., 2004). These observations suggested the potential role of abnormal oscillatory activity in PD.

Further deeper research uncovered a fascinating phenomenon known as phase-amplitude coupling (PAC) within the STN (Brittain and Brown, 2014). PAC describes a specific interaction, in which the amplitude of a high-frequency oscillation (HFO) is modulated by the phase of a lower-frequency oscillation (e.g., beta). PAC is thought to reflect synchronization of distinct neuronal populations and is a potential underlying mechanism of information processing and communication within the basal ganglia (Boraud et al., 2005). Studies have demonstrated that patients with PD exhibited excessive coupling between beta activity (13–30 Hz) and high-frequency gamma activity (60–100 Hz), particularly within the motor cortex (de Hemptinne et al., 2013; Gong et al., 2021, 2022) and STN (Baker Erdman et al., 2025; Yang et al., 2014). This abnormal PAC was found to correlate with bradykinesia and worsening in transitions between movement states, with higher coupling values associated with greater motor impairments, and has been implicated in the pathophysiology of PD (Baker Erdman et al., 2025; Gong et al., 2021; van Wijk et al., 2016; Yang et al., 2014). Moreover, excessive PAC was identified as a potential biomarker of motor impairment in PD and has been linked with locking of neural spiking activity to beta oscillations within the STN (Meidahl et al., 2019). This locking mechanism is believed to be the basis of the motor symptoms, such as rigidity and tremors, in PD. In particular, PAC was demonstrated to be specific for PD, based on previous results that it was significantly higher in patients with PD than in those with other movement disorders (compared with beta power, which was also exaggerated in dystonia and essential tremor) (Baker Erdman et al., 2025; de Hemptinne et al., 2013).

Dopaminergic medications, such as levodopa, dramatically influence the dynamics of PAC. Studies demonstrated that dopaminergic treatment mitigated excessive PAC in off states, suggesting its beneficial effects in regulating oscillatory patterns in motor symptoms (T＀＀Bocci et al., 2024a, Bocci et al., 2024b; Farokhniaee et al., 2023; Zhao et al., 2025). Initiation of dopaminergic therapies was observed to shift coupling dynamics. For example, Iskhakova et al. demonstrated that modulation of dopamine levels led to frequency shifts in cortico-basal ganglia beta oscillations, facilitating better neuronal firing profile and influencing PAC patterns (Iskhakova et al., 2021).

DBS is another crucial method to alter PAC in PD. Evidence showed that therapeutic DBS effectively reduced abnormal PAC, especially in the motor cortex, leading to improved motor function and reduced rigidity (de Hemptinne et al., 2015; Salimpour et al., 2022). Furthermore, Kato et al. highlighted changes in PAC dynamics based on voluntary muscle contractions during stimulation, indicating the potential for personalized treatment in PD (Kato et al., 2016). Movement execution also modifies PAC dynamics. Gong et al. demonstrated that PAC increased during repetitive movements in patients with PD and illustrated movement-related changes in oscillatory coupling, which corresponded with performance deficits (Gong et al., 2022).

Another interesting aspect of PAC would be the relationship between ongoing STN activity and tremor dynamics, as measured by concurrent electromyography (EMG) activity. For example, Kumaravelu et al. recorded LFPs from DBS electrodes in the thalamus and found that spectral power, especially in the theta band, exhibited strong correlation with tremor signals on accelerometers or EMG (Kumaravelu et al., 2025). In the STN, gamma oscillations (30–100 Hz) have been particularly associated with tremor activity. Beudel et al. reported that tremor reduction by DBS corresponded with gamma power suppression in the STN, reinforcing that low gamma activity is intricately linked with tremor expressions in patients with PD (Beudel et al., 2015). Furthermore, Weinberger et al. identified that increased STN gamma activity correlated with tremor episodes and indicated a direct relationship between these oscillatory patterns and movement disorders (Weinberger et al., 2009).

Although there have been some studies investigating these relationships, no direct investigation on the relationship between the tremor phase and the power of ongoing STN oscillation has been reported. Historical research by Reck et al. characterized the distinct LFP signals during different tremor states (Reck et al., 2010). Their analysis focused on the coherence between STN LFPs and EMG of the muscles involved in movements and provided evidence of how these neural signals manifested symptoms of tremor at the muscular level.

Furthermore, the relationship between PAC and clinical severity of PD, as measured by standardized scales, such as the Movement Disorder Society Unified Parkinson's Disease Rating Scale (MDS-UPDRS) (Goetz et al., 2008), has not been extensively explored. Identifying potential correlations between PAC and MDS-UPDRS scores could offer a novel approach to objectively assess disease severity and treatment efficacy. Although the available studies have established that medication and movement independently influence PAC in PD, the distinct contributions of each have not been systematically investigated in detail. Most studies investigated the effect of medication or movement, leaving a critical gap in the understanding of how these variables interact. Moreover, most of the research on PAC modulation by medication and movement have relied on cortical recordings, such as electroencephalogram (EEG), and only few studies systematically investigated these dynamics directly from the STN.

The primary objective of this study was to systematically differentiate the independent and interactive effects of dopaminergic medication (levodopa) and motor state (static vs. kinetic) on PAC within the STN. A second key objective was to determine the clinical relevance of these oscillatory dynamics by exploring the relationship between STN PAC measures and motor impairment, as assessed by the MDS-UPDRS. Furthermore, we aimed to investigate the coupling between peripheral tremor dynamics and central STN oscillations by quantifying PAC between the tremor-related EMG signal phase and the STN activity power in different frequency bands. Our hypothesis was that pathological beta-HFO PAC within the STN would be highest in the off-medication state, particularly during kinetic tasks, and would be significantly suppressed by levodopa. We further hypothesized that a significant coupling exists between the peripheral tremor phase (EMG) and power of STN oscillations and that STN-EMG coupling would be strongest in the untreated state and can modulated by medication. Finally, we aimed to determine the correlation of the magnitude of these pathological PAC measures with clinical motor severity, as indicated by higher MDS-UPDRS-III scores.

## 2 Materials and methods

### 2.1 Participants

This study analyzed the publicly available dataset at openneuro.org. The experimental protocol was described extensively in the article that primarily published the dataset (Rassoulou et al., 2024). All included patients (n = 20) were diagnosed with idiopathic PD and underwent DBS surgery on the day before measurement. A summary of the demographic data of the participants is listed in Table 1. Detailed descriptions of the dataset and study protocol may be referred to in the published dataset. The study was approved by the ethics committee of the Medical Faculty of the Heinrich Heine University Düsseldorf (Study No. 3209); it was carried out in accordance with the Declaration of Helsinki and required written informed consent.

**Table 1:** Demographics of the study population.

Sex	n	Age (mean ± SD)	Laterality (L/R/B)	Disease duration (mean ± SD)	MDS-UPDRS OFF (mean ± SD)	MDS-UPDRS ON (mean ± SD)
M	14	62 ± 8.3	8/3/3	9.2 ± 4.2	36.7 ± 11.1	22.3 ± 10.5
F	6	64.5 ± 7.6	4/2/0	12.3 ± 5.7	39.3 ± 10.8	23 ± 9.1

### 2.2 Recording setup

The study parameters were recorded from patients one day after electrode implantation to the STN, with leads still externalized. In almost all patients, recordings were performed in two sessions: 1) when patients were off oral dopaminergic medication for at least 12 h and 2) when they were on medication; in one patient, only the off-medication session was recorded. Subcutaneous apomorphine administration was paused 1.5–2 h before the measurements. Each session had two movement conditions, including 5 min of rest followed by static forearm extension (hold) and 5 min of rest followed by self-paced fist clenching (move). There were pauses between movements. In some patients, recordings were made in the resting state only (rest).

LFPs from the STN, the magnetoencephalogram and the surface EMG of the extensor digitorum communis and flexor digitorum superficialis muscles of both upper limbs were recorded simultaneously. The sampling rate was 2000 Hz. DBS electrodes were connected to the MEG system amplifier using externalized nonferromagnetic leads. Electrode contacts were referenced to the left mastoid and rearranged to a bipolar montage offline by subtracting signals from neighboring contacts. EMG electrodes were referenced to surface electrodes on the muscle tendons. A hardware filter for the EMG was applied using a passband of 0.1–660 Hz.

### 2.3 Analyses

#### 2.3.1 Preprocessing

LFP artifacts were identified as periods with strong broadband modulation of the signal. To identify good contact points of the DBS electrode, we calculated the LFP signal power during medication off and on periods in the beta frequency range. If the signal power during the off period was greater than the mean +2SD power during the on period, the contact point was labeled as good. This criterion was chosen to ensure the analysis focused on contacts exhibiting the pathological beta reactivity characteristic of the dorsolateral 'motor' STN (Weinberger et al., 2006). The +2SD threshold corresponds to the standard statistical convention (approx. 95th percentile) used to distinguish significant signal deviations from a baseline distribution, similar to methods used for detecting oscillatory bursts (Tinkhauser et al., 2017). Only these contact points were used in the later analysis.

#### 2.3.2 Cross-frequency coupling

Although cross-frequency coupling (CFC) comes in several forms (Jensen and Colgin, 2007), we used PAC within the STN. The calculation followed a previously described procedure (Cohen, 2008, 2014). First, the raw LFP signal from one channel was band-pass filtered to a high-frequency band. The power time series of the previously filtered signal was then calculated using the Hilbert transform (a). The raw analytical signal of the same channel was then band-pass filtered to a lower-frequency band, and the phase time series was calculated again using the Hilbert transform on the filtered signal (ɸ). The PAC was calculated using the following equation suggested in earlier studies (Canolty and Knight, 2010; Cohen, 2014):

$$PAC=\left|n^{-1}\sum_{t=1}^{n}a_{t}e^{i\left({ɸ}_{t}-\bar{ɸ}\right)}\right|$$

where t is the time point, a is the power of HFO at time point t, i is the imaginary operator, ɸ is the phase angle of beta at time point t, and n is the total number of time points; n was corrected using a debiasing factor $\underline{ɸ}$, as previously suggested (van Driel et al., 2015).

To avoid different confounding factors for PAC, nonparametric permutation tests for deviations from the null hypothesis distribution were applied (Cohen, 2014). The null hypothesis distribution was calculated the same way as PAC, but the power time series of the filtered high-frequency signal was iteratively time-shifted. For each bin of the comodulogram, PACz was calculated as the z-value between the generated null hypothesis distribution and PAC; the frequency was 4–40 Hz for phase and 200–400 Hz for amplitude. The filtering bandwidth was different for the lower and higher frequencies. For the lower frequencies (frequency for phase), the bandwidth was 3 Hz and centered around the given low frequency. For the higher frequencies (frequency for amplitude), the bandwidth was set at ±0.4∗fx around the given high-frequency fx.

We grouped PACz results in a two-way setup: medication on/off and task (rest/move/hold), according to the side of the task. For example, if the task was to hold the left arm, the LFP recorded from the contralateral STN was analyzed. If the task was to rest, we analyzed both STN signals. For statistical analysis, we calculated the maximum PACz of the comodulogram. We performed two-way analysis of variance (ANOVA) on factors during medication on/off and task (rest/move/hold). Posthoc Tukey HSD test was performed to evaluate significant differences.

#### 2.3.3 Correlation between PACz and MDS-UPDRS III

To determine the relationship between PAC strength and clinical symptoms, we calculated the Pearson correlation coefficient between each individual PACz matrix and different MDS-UPDRS scores, which were grouped into different subscores to account for different pathophysiologic mechanisms, as follows.

(1) Axial-related scores (MDS-UPDRS 3.10–3.13) and PACz calculated within the contralateral STN during movement,
(2) Scores related with akinesia-bradykinesia (MDS-UPDRS 3.4–3.8) and calculated PACz within the contralateral STN during movement,
(3) Scores related with kinetic tremor (MDS-UPDRS 3.16) and calculated PACz within the contralateral STN during movement,
(4) Scores related with postural tremor (MDS-UPDRS 3.15) and calculated PACz within the contralateral STN during the hold condition, and
(5) Scores related with resting tremor (MDS-UPDRS 3.17) and calculated PACz within the contralateral STN during rest.

The correlation coefficient between each selected MDS-UPDRS score and PACz contralateral to the selected score was calculated in each bin of the comodulogram. The correlation coefficients were corrected for multiple comparisons using permutation testing.

#### 2.3.4 EMG-STN PAC

To investigate the relationship between movement and tremor kinetics, we calculated PACz, as described earlier, in the EMG signal phase at the tremor frequency (4–8 Hz) and the STN signal power at beta and HFO (200–400 Hz). Prior to analysis, the absolute value of the raw EMG signal amplitude on the limb of the more affected side, (based on the demographics), underwent full-wave rectification to provide a more direct estimate of the muscle activation envelope (Merletti and Parker, 2004).

The preprocessed EMG was first band-pass filtered at the typical tremor frequency range (4–8 Hz), and its instantaneous phase (ɸ) was extracted using the Hilbert transform. Concurrently, the raw LFP signal of the contralateral STN was band-pass filtered into two distinct bands of interest: beta (13–25 Hz) and HFO (200–400 Hz). The power of each STN frequency band was extracted using the Hilbert transform. Finally, PACz was calculated between the EMG phase and STN power for both beta and HFO bands using the method described previously.

Therefore, within the formula we presented earlier for the calculation of EMG-STN PAC “a” denoted the ongoing STN power of a specific band (beta or HFO), and “ɸ” denoted the EMG signal phase filtered at 4–8 Hz. Similar to CFC calculation in the STN, we grouped PACz results in a two-way setup: medication on/off and task (rest/move/hold), then performed two-way ANOVA.

One participant was excluded from the repeated-measures ANOVA (n = 19 for this specific analysis) to satisfy the requirement for complete within-subject datasets, as this subject had only medication “OFF” state sessions.

## 3 Results

After preprocessing the data, we identified 35 good contact points in the left STN and 36 in the right STN (See Supplementary material). The results presented here were based on these contact points.

### 3.1 Cross-frequency coupling in the STN

**Fig. 1:**
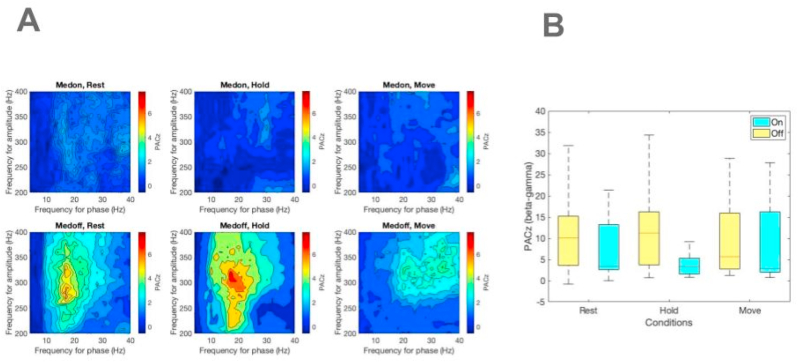
Phase-amplitude coupling within the STN (A) The average comodulograms of the patients in medication on (upper row) and medication off (lower row) and rest/hold/move conditions (columns) are shown. The coupling strength, which is measured as Z-score (from 0 to 7), is the same among the comodulograms, as denoted by the color scale. (B) The distribution of the peak PACz in each dataset under different conditions is shown.

We observed an increase in CFC between the 13–20 Hz phase and power of oscillations >250 Hz during the off period (Fig. 1; effect of medication: F = 4.1 p = 0.04, ηp^2^ = 0.19). We did not find any significant effect of the concurrent task (effect of task: F = 0.52, p = 0.59, ηp^2^ = 0.03; interaction between task and medication: F = 0.47, p = 0.62, ηp^2^ = 0.03). Posthoc analysis revealed a significant difference between off and on medication states during rest and hold conditions but not during the move condition. The descriptive statistics for each condition are shown in Table 2.

**Table 2:** Descriptive statistics of the maximum beta-HFO coupling strength under each condition.

Medication	Task	median	SD	IQR
OFF	Rest	10.06	2.25	11.64
	Hold	11.22	2.4	12.5
	Move	5.65	2.31	13.17
ON	Rest	3.38	2.1	10.62
	Hold	3.35	2.07	3.73
	Move	2.84	2.34	14.19

### 3.2 Correlation between PACz and MDS-UPDRS III

**Fig. 2:**
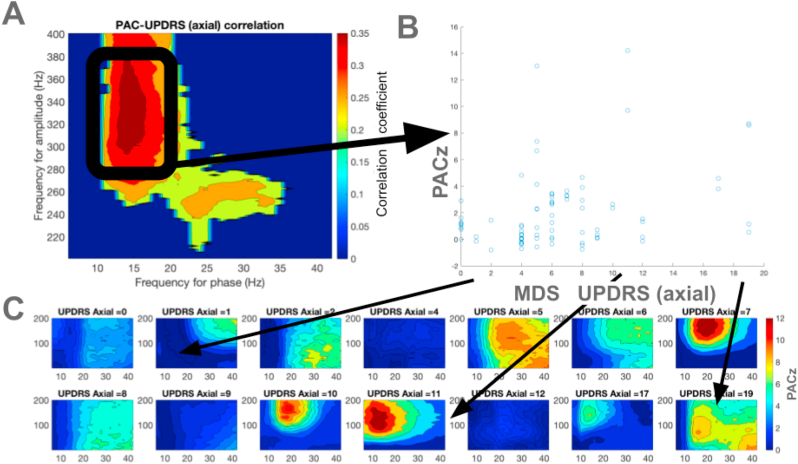
Correlation between phase amplitude strength and MDS-UPDRS axial scores (A) The correlation coefficients between coupling strength and MDS-UPDRS axial scores of each registration in the comodulogram are shown. (B) The relationship between average coupling strength in the selected window for each registration with MDS-UPDRS axial scores are shown. (C) The average PACz comodulograms at each MDS-UPDRS axial score are shown; the color scales are identical among the comodulograms.

**Fig. 3:**
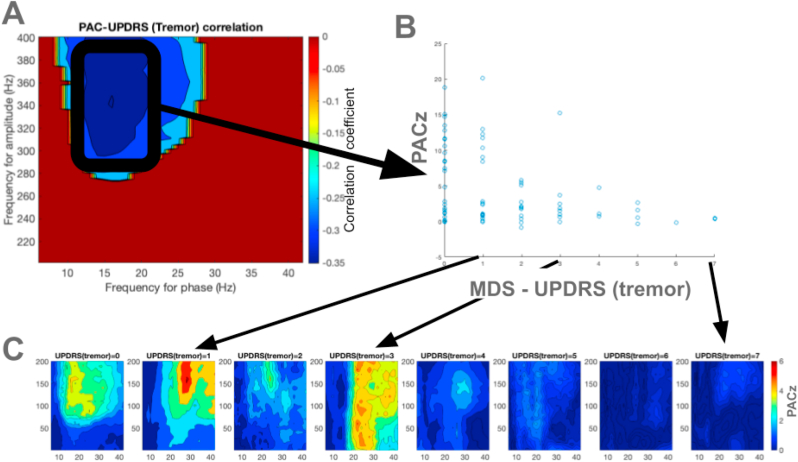
Correlation between phase amplitude strength and MDS-UPDRS tremor scores (A) The correlation coefficients between coupling strength and MDS-UPDRS tremor scores of each registration in the comodulogram are shown. (B) The relationship between average coupling strength in the selected window for each registration with MDS-UPDRS tremor scores are shown. (C) The average PACz comodulograms at each MDS-UPDRS tremor score are shown; the color scales are identical among the comodulograms.

**Fig. 4:**
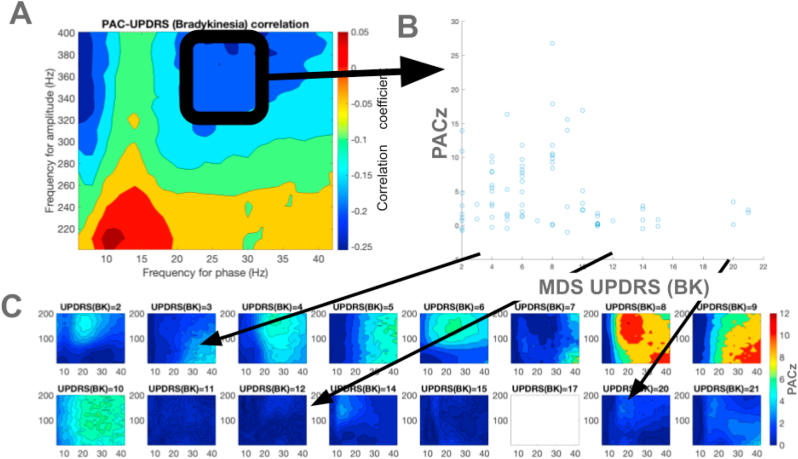
Correlation between phase amplitude strength and MDS-UPDRS BK scores (A) The correlation coefficients between coupling strength and MDS-UPDRS BK scores of each registration in the comodulogram are shown. (B) The relationship between average coupling strength in the selected window for each registration with MDS-UPDRS BK scores are shown. (C) The average PACz comodulograms at each MDS-UPDRS BK score are shown; the color scales are identical among the comodulograms.

We observed a significant positive correlation between the MDS-UPDRS axial scores and strength of the beta (13–20 Hz)-HFO (200–400 Hz) PAC, with a median Pearson's rho of 0.35 in the selected window (p < 0.01, Fig. 2). There was a significant negative correlation between the strength of the beta (13–20 Hz)-HFO (200–400 Hz) PAC and MDS-UPDRS scores related with tremor, with a median Pearson's rho of −0.32 in the selected window (p < 0.01, Fig. 3). However, we did not observe any significant correlation between coupling strength and MDS-UPDRS scores related with akinesia-rigidity (Fig. 4). Given the sample size (n = 20) and the exploratory nature of the tremor correlation, these findings should be interpreted with caution.

### 3.3 STN-EMG PAC

We found no significant PAC between the phase of low frequency oscillations in the limb (around the tremor frequency) and the power of HFO (both in beta and >200 Hz oscillations) in the STN (Supplementary material). In addition, we found no significant differences between the conditions (effect of task: F = 0.25, p = 0.7; effect of medication: F = 0.3, p = 0.67; interaction: F = 0.22, p = 0.79).

## 4 Discussion

In this study, we investigated the effects of dopaminergic modulation and different kinetic conditions on PAC within the STN. Overall, we observed increased beta-HFO PAC during the medication off period in the resting and hold conditions but not during the movement condition. We also found that PAC strength was correlated with the severity of symptoms, as measured by the MDS-UPDRS scores.

The most striking result of this study was the higher in PAC during the levodopa off period than during the on period, particularly in resting and hold conditions. This observation aligned with previous studies, which demonstrated elevated beta and gamma/HFO power in the STN during the off state (Alonso-Frech et al., 2006; Weinberger et al., 2006; Yang et al., 2014). Enhanced PAC suggests greater synchronization between beta and gamma/HFO oscillations in the absence of dopaminergic stimulation. This potentially reflects a compensatory mechanism within the basal ganglia to maintain functionality despite depleted dopamine levels (Rivlin-Etzion et al., 2006; Weinberger et al., 2006) and aligns with the known effects of dopaminergic medication on basal ganglia oscillations. Several studies reported that levodopa administration decreased the power and frequency of beta oscillations to levels closer to those observed in healthy individuals (Gulberti et al., 2015; Iskhakova et al., 2021; Ramirez Pasos et al., 2019) and that dopamine tone adjusted beta oscillation frequency, initially shifting it upward and later returning it to baseline as the medication effect waned (Iskhakova et al., 2021).

In addition to CFC modulation, reconfiguration of the internal structure of HFOs themselves has been shown to be an effect of dopaminergic medications. Several studies reported that levodopa intake increased the power ratio of fast HFOs (fHFO, 300–400 Hz) relative to slow HFOs (sHFO, 200–300 Hz), suggesting this spectral shift as a prokinetic signal (Foffani et al., 2003; Kane et al., 2009; Özkurt et al., 2011). Although our analysis did not directly analyze a shift in this sHFO/fHFO power ratio, we did confirm robust attenuation of the overarching beta-HFO PAC. These two phenomena may be mechanically linked. It was plausible that the pathological beta phase coupling dominance in the off state preferentially trapped or entrained oscillations within the sHFO band. By attenuating this pathological beta phase lock, dopaminergic medication may release sHFO activity, thereby, permitting the high-frequency network to reorganize into a more physiological state, which is characterized by a relative increase in fHFO power. Therefore, suppression of beta-HFO PAC, which was a clear and significant finding in our study, could be the primary mechanism that facilitates spectral HFO reorganization.

In contrast to the clear effect of the medication at rest, the lack of significant differences in PAC during movement in the off state warrants further exploration. Possibly, movement itself disrupts the synchronized activity reflected in PAC or the increased effort required for movement in the off state masked any underlying differences. Indeed, previous research indicated that participation of patients in motor activities resulted in beta band suppression and enhanced gamma oscillatory activity in the STN, leading to improved motor performance (Gong et al., 2022; Ramirez Pasos et al., 2019). Furthermore, increased beta synchronization before movement can hinder this process and ultimately affect movement amplitude and velocity, which are commonly observed characteristics in PD (Chen et al., 2019; Singh et al., 2013; Toledo et al., 2014).

Beta band oscillations (13–30 Hz) within the STN have been shown to be tightly coupled with HFOs (200–500 Hz), indicating abnormal PAC as a hallmark of the pathophysiological state in PD (van Wijk et al., 2016; Yang et al., 2014). Yang et al. highlighted that increased PAC was associated with worse motor performance and indicated that beta-coupled HFOs may compromise functional adaptability of the motor circuit in PD (Yang et al., 2014).Beyond the effects of medication and movement, a key objective of our study was to relate these oscillatory dynamics with clinical symptoms. The positive correlation between PAC strength and bradykinesia MDS-UPDRS score in this study was particularly interesting. This finding suggested that PAC may reflect the underlying neurophysiological processes that contribute to the motor symptoms of PD. Indeed, earlier studies reported that pathological synchronization of beta (13–30 Hz) oscillations in the basal ganglia was linked with motor symptoms, indicating that excessive beta activity correlated with increased severity of bradykinesia and rigidity in patients with PD (Baker Erdman et al., 2025; Muthuraman et al., 2021; Ray et al., 2008). Specifically, abnormal beta oscillatory dynamics have been associated with dysregulated motor control, and bradykinesia (i.e., slow movement and difficulty initiating motion) was particularly influenced by alterations in beta–gamma PAC (Hodnik et al., 2024; Tinkhauser et al., 2018).

The coupling dynamics between the STN and tremor-producing oscillations has been a particular focus of research. Hirschmann et al. identified a direct correlation between STN–cortex PAC and rest tremor frequency and suggested that heightened coupling during tremor epochs could contribute to the persistence of motor symptoms (Hirschmann et al., 2013). Notably, the enhanced coupling between the STN beta frequency oscillations and tremor frequencies during the off-medication state supported the need for effective interventions that target these maladaptive couplings to relieve tremor-associated disabilities (Van Wijk et al., 2017). However, an intriguing aspect of our results was the relationship between STN PAC and parkinsonian tremor. The significant negative correlation between beta-HFO PAC and MDS-UPDRS tremor subscores suggested that higher levels of this specific coupling within the STN are associated with less severe tremor. This seemingly counterintuitive finding aligned with the growing consensus that tremor and akinetic-rigid symptoms may arise from distinct and, perhaps, competing pathophysiological networks (Helmich, 2018; Helmich et al., 2021). Although excessive beta band activity and its associated PAC have been strongly linked with bradykinesia and rigidity (Chen et al., 2019; Singh et al., 2013; Toledo et al., 2014), the tremor network is thought to be driven by oscillations in lower-frequency bands, such as the theta range (Contreras-Vidal and Stelmach, 1995; Helmich, 2018). Therefore, our results could have reflected a state-dependent tradeoff, in which the basal ganglia network dominated by a pathological beta-HFO PAC state may have been less permissive to tremor generation and vice-versa. This interpretation was further supported by our inability to find any direct PAC between the peripheral tremor phase measured by EMG and the amplitude of ongoing STN oscillations. However, it would be crucial to acknowledge that this result does not preclude the existence of other forms of neural coupling. Methodologically, our analysis focused on cross-frequency PAC, whereas another study successfully used coherence to demonstrate a linear phase relationship between STN and EMG signals at the tremor frequency (Reck et al., 2010). The absence of PAC in our data, therefore, does not rule out a relationship but rather suggested that the specific mechanism of beta phase entrainment of high-frequency activity is not the primary driver of tremor generation. However, we acknowledge that our sample size (n = 20) limits the statistical power to detect weaker couplings, and this absence of evidence should not be interpreted as definitive evidence of absence.

The potential utility of PAC includes its clinical correlations and dynamic nature. DBS for PD targets the STN, with beta power often used for electrophysiological localization during electrode implantation (Chen et al., 2006; Hamani et al., 2004; Holdefer et al., 2010). However, beta power alone may not fully capture the complexities of abnormal neuronal activity in PD. PAC, which reflects synchronization between beta and gamma oscillations, may provide complementary information regarding the dysfunctional network dynamics within the basal ganglia (Baker Erdman et al., 2025; de Hemptinne et al., 2013). This dynamism further strengthens the potential of PAC as a biomarker for adaptive DBS. Unlike beta power, which may remain relatively constant, PAC could offer real-time feedback on the state of the basal ganglia circuitry. Future studies could investigate if this information can inform stimulation parameters in real-time, thereby, tailoring therapy based on the needs of each patient and disease fluctuations (Little and Brown, 2020). For example, during off periods when PAC is elevated, increased stimulation intensity could be delivered to counteract excessive synchronization and improve motor function (Buhmann et al., 2015). Conversely, during on periods or movement states, when PAC may be lower, stimulation intensity could be reduced to minimize side effects. This adaptive approach holds promise for optimizing therapeutic benefits, while minimizing the risk of DBS-induced complications (Priori et al., 2021).

Although this study offered valuable information, certain limitations must be acknowledged. A key limitation of this study was the timing of assessments during the acute postoperative phase (i.e., one day after DBS implantation). This timing might have introduced two major confounding factors. First, electrode insertion may have caused a stunning effect on microlesions and temporarily improved clinical symptoms, thereby, masking the true baseline state of the patient. Second, the LFP signal itself was likely affected by acute physiological reactions, such as perilead edema and inflammation, after surgery. These factors can alter the electrode–tissue interface and distort the recorded LFP properties, such as power in the specific frequency bands. Consequently, both the clinical and neurophysiological data collected at this early time point may not be representative of the patients’ stable chronic condition; therefore, all results must be interpreted with caution. In addition, the small sample size restricted the statistical power and generalizability of the results. The cohort was treated as a single group without differentiating between the motor phenotypes of PD (e.g., tremor-dominant vs. akinetic-rigid); this design may have obscured phenotype-specific findings. Finally, the cross-sectional design of this study at a single acute time point inherently limited any causal inference and precludes understanding of the evolution of these relationships in the chronic state.

Despite these limitations, this work characterized beta-HFO PAC within the STN as a specific and dynamic biomarker that reflected the dopaminergic state and clinical phenotype of patients with PD. The findings can help deepen our mechanistic understanding of basal ganglia pathophysiology and provided a clear translational pathway toward the development of more sophisticated and personalized neuromodulation therapies.

## 5 Conclusions

This study provided new evidence for the dynamic nature of PAC within the STN in PD. Its modulation by the dopaminergic state and potential correlation with MDS-UPDRS scores suggested that beta-HFO PAC may serve as a valuable tool for understanding and managing PD. Future research based on these findings has the potential to revolutionize our approach to diagnose, monitor, and treat this debilitating disease.

## Ethical compliance statement

Data collection was approved by the Ethics Committee of the Medical Faculty of Heinrich Heine University Düsseldorf (study no. 3209). Informed consent was obtained from each participant. Participants' consent to the open publication of the anonymized data was waived by the Data Protection Office of University Clinic Düsseldorf. We confirm that we have read the Journal's position on issues involved in ethical publication and affirm that this work is consistent with those guidelines.

## Funding statement

This research received no specific grant from any funding agency in the public, commercial, or not-for-profit sectors.

## CRediT authorship contribution statement

**András Puszta:** Conceptualization, Formal analysis, Methodology, Project administration, Visualization, Writing – original draft. **Dénes Zádori:** Conceptualization, Supervision, Writing – original draft, Writing – review & editing. **Péter Klivényi:** Conceptualization, Supervision, Writing – review & editing.

## Declaration of competing interest

The authors declare that they have no known competing financial interests or personal relationships that could have appeared to influence the work reported in this paper.

The author is not an Editorial Board Member/Editor-in-Chief/Associate Editor/Guest Editor for *Neuroimage: Reports* and was not involved in the editorial review or the decision to publish this article.

## Data Availability

The data used for the publication is openly available and cited in the manuscript.
