# Supplementary material for: Increased beta-HFO phase-amplitude coupling in the subthalamic nucleus during movement in Parkinson's disease
Source: Neuroimage Rep. 2026 May 13;6(2):100353. doi: 10.1016/j.ynirp.2026.100353 (PMC13196517; doi:10.1016/j.ynirp.2026.100353)
Supplement: Multimedia component 1 [file mmc1.docx]

Supplementary material: Increased Beta-HFO Phase-Amplitude Coupling During Movement in Parkinson's Disease.

**Authors list:**

András Puszta MD, PhD ^1,2,3*^

Dénes Zádori MD, PhD^1^

Péter Klivényi MD, PhD^1,4^

^1^: University of Szeged, Department of Neurology

^2^: Helgeland Hospital, Department of Neuropsychology

^3^: University of Oslo, Department of Psychology

^4^: HUN-REN-SZTE Neuroscience Research Group, Hungarian Research Network, University of Szeged

*: Corresponding author e-mail address: puszta.andras@med.u-szeged.hu

# Good contact points:

| ID | Left | Right |
| --- | --- | --- |
| sub-0cGdk9' | [1;0;1] | [1;1;1] |
| sub-2IU8mi' | [0;0;1] | [0;1;0] |
| sub-2IhVOz' | [1;1;1] | [1;1;1] |
| sub-6m9kB5' | [0;0;0] | [0;0;0] |
| sub-8RgPiG' | [1;1;0] | [1;1;1] |
| sub-AB2PeX' | [1;1;1] | [0;0;0] |
| sub-AbzsOg' | [1;1;1] | [0;0;0] |
| sub-BYJoWR' | [0;1;1] | [1;1;1] |
| sub-BgojEx' | [;1;1;1] | [1;1;1] |
| sub-FIyfdR' | [0;1;0] | [0;0;0] |
| sub-FYbcap' | [0;1;1] | [1;1;1] |
| sub-PuPVlx' | [0;0;0] | [0;0;0] |
| sub-QZTsn6' | [0;1;1] | [1;1;1] |
| sub-VopvKx' | [1;1;1] | [1;1;0] |
| sub-dCsWjQ' | [0;1;0] | [1;1;1] |
| sub-gNX5yb' | [0;0;0] | [0;0;0] |
| sub-hnetKS' | [1;1;1] | [1;1;0] |
| sub-i4oK0F' | [0;1;1] | [0;0;0] |
| sub-iDpl28' | [1;0;0] | [1;0;0] |
| sub-jyC0j3' | [1;0;0] | [1;1;1] |
| sub-oLNpHd' | [0;1;1] | [0;0;1] |
| sub-zxEhes' | [0;0;0] | [0;0;0] |

Supplementary table 1: Indices of the good contact points within each subject/recordings in the dataset. Zero denotes bad contact point, 1 denotes good contact point.

2. EMG-LFP phase amplitude coupling
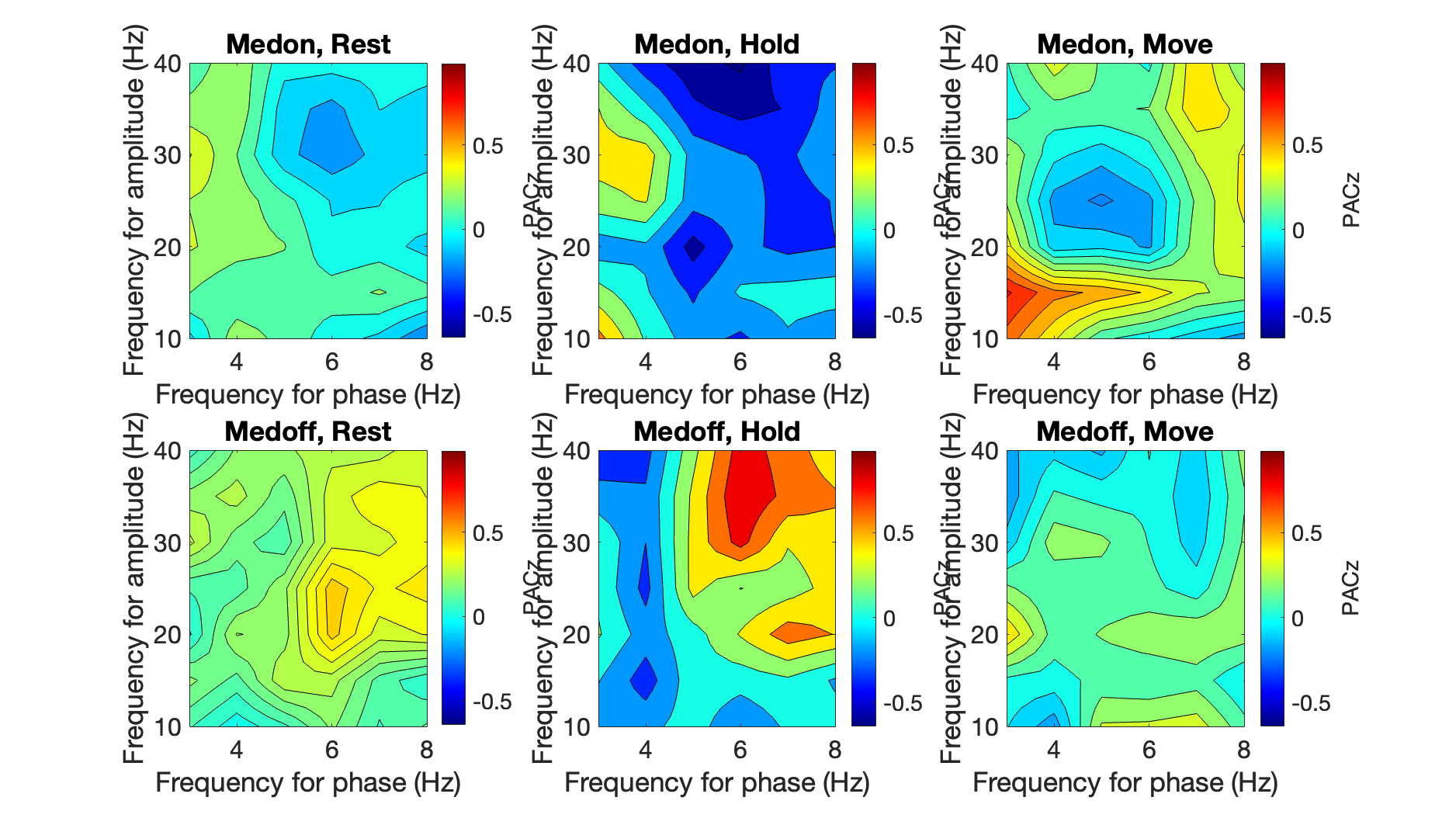


*Supplementary Figure 1: Phase-amplitude coupling between limb EMG phase at tremor frequency and the amplitude of 10-40 Hz in the contralateral STN in different conditions. Comodulograms in medication ON (upper row) and medication OFF (lower row) in the three different conditions (Rest/Hold/Move) in the three columns, respectively. The color-scale denotes the coupling strength measured in Z-score (from 0 to 7) and it is the same across the comodulograms. Note, that while there are seemingly different phase-amplitude coupling patterns in the comodulograms, none of the coupling strength was significant (i.e., z score of the PAC < 1)*


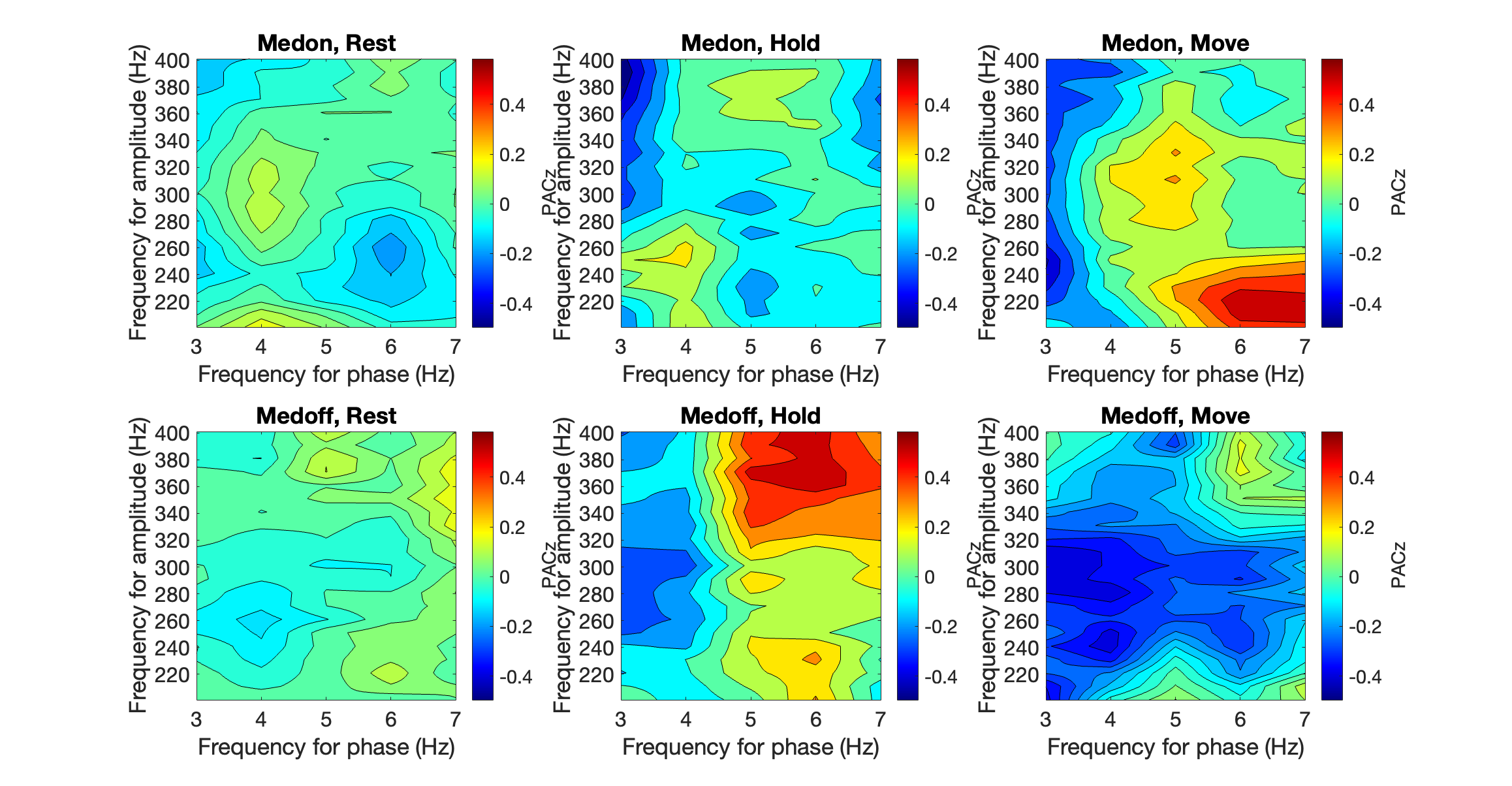


*Supplementary Figure 2: Phase-amplitude coupling between limb EMG phase at tremor frequency and the amplitude of 200-400 Hz in the contralateral STN in different conditions. Comodulograms in medication ON (upper row) and medication OFF (lower row) in the three different conditions (Rest/Hold/Move) in the three columns, respectively. The color-scale denotes the coupling strength measured in Z-score (from 0 to 7) and it is the same across the comodulograms. Note, that while there are seemingly different phase-amplitude coupling patterns in the comodulograms, none of the coupling strength was significant (i.e., z score of the PAC < 1)*
